# Unique features of Ayurveda dietetics

**DOI:** 10.1186/1878-5085-5-S1-A165

**Published:** 2014-02-11

**Authors:** Naushad Ali Tachaparamban

**Affiliations:** 1 Atreya Ayurveda, Moscow, Russia

## 

Ayurveda has a personalized approach on dietetics. Requirement of food intake is assessed on the basis of constitution in healthy individuals. In an ideal condition instinct of the subject is the indicator of bodies needs to compensate deficient dosha. Assessment of quality of food is made subjectively through six tastes, attributes, potency and metabolized taste. Ayurveda gives more importance in regulates the digestive power than correction of the calorie of food. According to the change in space and time dosha predominance varies. This warranted a consideration in choosing the right diet. Ayurveda emphasized the need of healthy mind for the proper nourishment of body. Various processing methods alter the attributes of food. Hence the assessment of food quality is to be made at the time of dining. Ayurveda warns against the complications of food-food interaction due to wrong combination of good materials. Unhealthy diet practice is considered as one of the important reasons of disease. Hence the correction of diet itself is considered as treatment.

